# Cardiorespiratory Fitness and Body Composition in Postmenopausal Women

**DOI:** 10.2478/hukin-2014-0099

**Published:** 2014-11-12

**Authors:** Helena Moreira, Betânia Passos, Josiane Rocha, Vivianne Reis, André Carneiro, Ronaldo Gabriel

**Affiliations:** 1 Department of Sport Sciences, Exercise and Health, Research Center for Sports Sciences, Health and Human Development (CIDESD), University of Trás-os-Montes and Alto Douro, Vila Real, Portugal.; 2 Department of Physical Education and Sports and Distance Education Center, University of Montes Claros, Montes Claros, Brazil.; 3 Department of Primary Care Master’s Program and Physical Education and Sports, University of Montes Claros and Integrated Colleges Pythagoras, Montes Claros, Brazil.; 4 Department of Physical Education, Integrated College of North Mine, Montes Claros, Brazil.; 5 Department of Physical Education, Integrated College of North Mine and Sports and Distance Education Center, University of Montes Claros, Montes Claros, Brazil.; 6 Department of Sport Sciences, Exercise and Health, Centre for the Research and Technology of Agro-Environment and Biological Sciences (CITAB), University of Trás-os-Montes and Alto Douro, Vila Real, Portugal.

**Keywords:** maximal oxygen uptake, visceral adiposity, menopause

## Abstract

The object of the study was to analyze the relationship between aerobic fitness and body composition in postmenopausal women. We hypothesized that postmenopausal women that had higher adiposity had lower cardiorespiratory capacity, regardless of the characteristics of menopause. The sample included 208 women (57.57 ± 6.62 years), whose body composition and the basal metabolic rate were evaluated by octopolar bioimpedance (InBody 720) and the oxygen uptake by the modified Bruce protocol. Most of the sample showed obesity and a high visceral fat area. The visceral fat area and the basal metabolic rate explained 30% of the variation of oxygen uptake, regardless of age, time, nature or hormone therapy. The values of the latter variables were reduced in the presence of high central adiposity (−6.16 ml/kg/min) and the basal metabolic rate of less than 1238 kcal/day (−0.18 ml/kg/min). The women with oxygen uptake above 30.94 ml/kg/min showed lower values of total and central adiposity when compared with other groups. With an increase of aerobic fitness, there was a growing tendency of the average values of the soft lean mass index, with differences between the groups low-high and moderate-high. These results suggest worsening of the cardiorespiratory condition with an increase of central adiposity and a decrease of the BMR, regardless of age and menopause characteristics.

## Introduction

A decline in estrogen levels that follows menopause leads to an increase in total and central fat (Teede et al., 2010), and a decrease in bone mass and muscular strength (Messier et al., 2011). Those changes in body composition are compounded by reduced levels of habitual physical activity and a basal metabolic rate (Poehlman et al., 1995), which contribute to worsening of cardiovascular fitness and the quality of life at this stage in life known as climacteric. The acquisition of an android model of fat distribution has an important clinical meaning not only related to an increased metabolic (Teede et al., 2010) and inflammatory (Valentine et al., 2009) risk, but also the risk of osteoporosis (Rolland and Vellas, 2009). Several authors have documented a greater influence of obesity on reduced physical capacity of postmenopausal women PW) compared to sarcopenia (Rolland et al., 2009). Estrogen deprivation contributes to a reduction of nitric oxide and prostacyclin by the endothelium (Weiner et al., 1994) and to an increased migration and proliferation of smooth muscle cells in inner walls of blood vessels (Bhalla et al., 1997). Obesity, age, years since menopause and hypertension are strong predictors of metabolic syndrome in (PW).

Analysis of the relationship between body composition and aerobic fitness appears to be very important in the definition of exercise programs to improve physical fitness and functional autonomy of (PW). Presence of lower levels of adiposity and better cardiorespiratory fitness favor the bipedal locomotion of that population keeping more appropriate levels of insulin and glucose, and a better lipoprotein profile with fewer inflammatory occurrences (Pansini et al., 2008). According to literature, there are conflicting results about the relationship between these two components of physical fitness. While some authors mention that the evolution of maximal oxygen uptake (VO_2max_) is not dependent on body composition (Hollenberg et al., 2006), others point out that low aerobic fitness in PW tends to be associated with increased levels of total and central adiposity (Pansini et al., 2008) and lean mass reduction (Hagberg et al., 2000). Regarding the influence of menopausal characteristics, some studies report an improvement in hemodynamics and vasodilator capacity with hormone therapy (Mercuro et al., 2007), while others reveal no meaningful variation in VO_2max_ (Hagberg et al., 2000). This study was designed to analyze the relative weight of several variables of body composition in the variation of VO_2max_ of PW. Based on the definition of three groups of VO_2max_ (ml/kg/min) established in the study, the average values of those variables, as well as the characteristics of menopause (hormone therapy, nature of menopause and time of menopause) were compared.

## Material and Methods

### Participants

The sample was composed of 208 PW (age, 57.60 ± 6.62 years; body mass, 68.90 ± 11.59 kg; body height 155.09 ± 0.05 cm), 51 of them showing an induced menopause. The use of hormone therapy was documented in 94 participants and 56.3% of women exhibited menopause less than 10 years.

Participants were recruited through newspaper advertisements, television, radio, e-mail messages, and flyers distributed in the community. Their inclusion in the “Shape up during Menopause Study” was based on evaluation of their reproductive and medical history. To define the status of reproductive age a modified STRAW (Stage of Reproductive Aging Workshop) classification (Soules et al., 2001) was used.

Subjects were excluded according to the following criteria: (1) premature menopause, (2) severe liver, kidney or blood diseases; (3) existence of cardiovascular diseases (symptoms of angina pectoris or myocardial infarction in the last 3 months) or uncontrolled hypertension (systolic arterial pressure level higher than 200 mmHg and diastolic arterial pressure level higher than 105 mmHg); (4) use of blockers and antiarrhythmic agents; and (5) existence of skeletal muscle conditions, neuro-muscular and neuro-physiological diseases that might prevent their participation in physical exercises or might have symptoms aggravated by them.

This study was approved by the Portuguese Foundation for Science and Technology and by the University of Trás-os-Montes and Alto Douro, and written consent was obtained from all the participants prior to the commencement of the study.

### Anthropometry and body composition

Body height (BH) was measured with a stadiometer (Seca 220, Seca Corporation, Hamburg, Germany) while body mass (BM), fat mass (FM), visceral fat area (VFA), skeletal muscle mass (SM), soft lean mass (SLM; total, arms, trunk and legs) and fat-free mass (FFM) were measured with octopolar bioimpedance InBody 720 (Biospace, Seoul, Korea). The validity of this bioimpedance has been documented in several studies (Malavolti et al., 2003; Medici et al., 2005; Ogawa et al., 2011). This technology employs eight contact electrodes, two are positioned on the palm and thumb of each hand and the other is placed on the front part of the feet and on the heels. It enables us to analyze five basic body parts, the left and right upper limb, trunk, and left and right lower limb, independently and using frequencies of 1, 5, 50, 250, 500 and 1000 kHz.

The basal metabolic rate (BMR) was calculated using the Cunningham’s equation (Cunningham, 1991) with measurements performed by the same technician in the morning and following standard methodology. The skeletal muscle mass index (SMI = SM/W ×100) was calculated according to Janssen et al. (2002) and the regional SLM (SLMI_A_, arms; SLMI_T_, trunk; SLMI_L_, legs) was adjusted to weight.

The points of reference for sarcopenia and obesity were, respectively, SMI ≤ 28% (Janssen et al., 2002) and FM ≥ 35% (Lohman and Going, 1998). Visceral fat values above 100 cm^2^ represent a strong risk for metabolic disturbances such as diabetes, hypertension and hyperlipidemia (Williams et al., 1996). Technical errors of variables (technical error = (∑*d*^2^/2*n*)^1/2^ where *d* is the difference between the evaluations and *n* is the sample size) were determined by two repeated measurements, in a subgroup of ten postmenopausal women (BH, 0.09 cm; BM, 0.06 kg; FM, 0.32 kg; VFA, 0.97 cm^2^; SM, 0.21 kg; SLM total, 0.35 kg; SLM right arm, 0.04 kg; SLM left arm, 0.04 kg; SLMT, 0.19 kg; SLM right leg, 0.03 kg; SLM left leg, 0.03 kg; FFM, 0.20 kg).

### Cardiorespiratory fitness

A submaximal test was conducted on a treadmill (Panatta Sport, Apiro, Italy) until 85% maximal heart rate was obtained, complying with the adapted Bruce protocol (Bruce et al., 1973). The expired gas was analyzed during implementation of the exercise protocol using the Sensormedics 2900 C gas analyzer (Sensor Medics Corporation, Yorba Linda, USA). The initial 3-minute stage occurred at a speed of 2.74 km/h and 0% gradient. The second and third stages had the same speed and duration, but the gradient was increased by 5% and 10%, respectively. Each subsequent stage had an increment of 1.29 km/h in speed and 2% in gradient. VO_2max_ was estimated using a linear regression of the mean heart rate and VO_2_ values of the last minute of each stage, between 55% and 85% of the predicted maximal heart rate. The classification of VO_2max_ was conducted in accordance with the criteria of McArdle et al. (2006) in five different levels (poor, fair, average, good and excellent), according to the age of the participants.

The women had been instructed to maintain their habitual medication and the following preparation guidelines were considered: refrain from consumption of alcohol, caffeine or tobacco products 12 hours before the test; use of comfortable sportswear; and absence of food intake 2 hours prior to testing.

### Statistical analysis

Data analyses were performed using a SPSS 16.0 package (SPSS Inc., Chicago, USA) and a p-value of ≤ 0.05 was considered for statistical significance. Data was expressed as mean ± standard deviation. The degree of association between variables was calculated by the Pearson’s correlation coefficient or Spearman’s correlation (characteristics of menopause). Multiple linear regression analysis (stepwise), controlling for age, BMR and characteristics of menopause were performed to evaluate associations between cardiorespiratory fitness and body composition. T-tests for independent samples were applied to compare groups, considering specific reference values: 100 cm^2^ (VFA) and 1238 kcal/day (BMR, value defined in this study). Considering three groups of VO_2max_ (<26.87; 26.87–30.94; >30.94), which were defined in order to obtain a number of equal (or approximately equal) observations within each group, comparison of the variables between the groups was made through the ANOVA or Kruskal–Wallis test (when the variables were not able to fill up all the assumptions for parametric statistics). The Bonferroni correction method was applied for multiple comparisons when necessary.

## Results

The age of the subjects ranged between 40.60 and 79.58 years (Table 1), and the mean values of % FM and FFM were, respectively, 39.34% and 41.17 kg, with 74% of the sample to reveal the presence of obesity (FM ≥ 35%). The vast majority of the sample presented an excess of visceral adiposity (85.6%) and a normal muscle condition (n = 191), and the average of BMR of 1238 kcal/day.

The largest amplitudes were found for regional SLM legs and trunk, representing these regions 17.41% and 28.64% of body mass, respectively. The average VO_2max_ recorded in the sample was 29.14 ml/kg/min, with most of the participants belonging to average (69.7%) and good (13.1%) levels and nineteen women presenting an excellent level. Cardiorespiratory fitness is influenced (p≤ 0.01) positively by the SMI (r = 0.48) and negatively by the presence of higher levels of total fat and especially by the VFA (r =− 0.53). Cardiorespiratory fitness tends to decrease with age and time elapsed since menopause, being particularly influenced by the first. Older women and those with longer amenorrhea reported a higher VFA (r = 0.37 and r = 0.32, respectively, p ≤ 0.01). Higher levels of MIG, SM and regional SLM (kg, trunk, arms, legs) were associated with increased energy expenditure.

The analysis of the influence of body composition on VO_2max_ (Table 2) showed that, regardless of age and characteristics of menopause, the VFA (β = −0,563, p ≤ 0.01) and BMR (β = 0,165, p ≤ 0.01) explained 30% of the variation in the dependent variable, with an estimated error of 4.76 ml/kg/min.

Considering the reference values established for the VFA and BMR (Table 3), postmenopausal women with the VFA ≥ 100 cm^2^ and BMR <1238 kcal/day showed the worst cardiorespiratory condition (p ≤ 0.01), translating into average values of −6.16 ml/kg/min and −0.18 ml/kg/min, respectively.

Table 4 shows the average values related to age, variables associated with body composition, characteristics of the menopause and BMR, depending on the reference values of VO_2max_ established in this study (LOW, <26.87; MODERATE, 26.87–30.94; HIGH >30.94). No differences were statistically significant for the characteristics of menopause and the BMR

The skeletal muscle mass index and regional SLMI (arms, legs and trunk) showed an increasing trend of average values of VO_2max_, reporting significant differences (p <0.01) between groups LOW-HIGH and MODERATE-HIGH, more marked for the SMI (−3.74% and −2.57%, respectively) and SLMIT (−2.56% and −1.78%). Compared to the levels LOW and MODERATE, women with VO_2max_ > 30.94 ml/kg/min had lower (p <0.01) FM (−6.72% and −4.28%, respectively) and the VFA (−29.42 cm^2^ and −14.49 cm^2^). Women with VO_2max_<26.87 ml/kg/min exhibited a higher VFA compared to those with a moderate level of cardiorespiratory fitness (−14.93 ml/kg/min, p<0.01). Cardiorespiratory fitness tends to decrease with age, with significant differences between groups LOW-MODERATE and LOW-HIGH.

## Discussion

As outlined in the introduction, previous this study aimed to analyze the relationship between body composition and cardiorespiratory fitness in postmenopausal women (40–79 years), exploiting the influence of several parameters in the variation of this latter variable, with the control of age, menopause characteristics and the BMR. Based on the established reference values for VO_2max_, we compared the average values of the variables related to body composition, TH, time and nature of the menopause. The results suggest that the VFA and BMR significantly influence the variation of cardiorespiratory fitness in PW, regardless of age and the characteristics of menopause. Women with levels of VO_2max_ > 30.94 ml/kg/min present lower total and central adiposity and better muscle condition in relation to other levels of cardiorespiratory fitness. Compared to moderate levels of cardiorespiratory fitness, the PW with VO_2max_ < 26.87 ml/kg/min are older and have greater intra-abdominal fat.

The chronic hypoestrogenism of these women was reflected in the changes of adipocytes metabolic properties. The most striking changes were an increase of visceral adiposity and reduction of the components of fat-free mass, regarding muscle and bone components in particular (Teede et al., 2010; Messier et al., 2011).

A very high percentage (85.6%) of women presented a VFA ≥ 100 cm^2^, revealing a higher risk to developing metabolic and endocrinal diseases, metabolic syndrome (Lemieux et al., 2011), osteoporosis and sarcopenia (Schrager et al., 2007). Since the role of central adiposity in the catabolism of proteins has been recognized (Schrager et al., 2007), derived in part from the decreased effect of insulin in protein synthesis and increased production of proinflammatory cytokines, the presence of a reduced number of women with sarcopenia may be linked to the average age of the sample (Rolland et al., 2008) and the characteristics of their menopause. A more pronounced decrease of estrogen and growth hormone in induced menopause (Greenlund and Nair, 2003) and a reduction of peripheral aromatization with the time of menopause (Szymczak et al., 1998) have been discussed in the literature. Other studies have indicated that central adiposity tends to increase with age and time since menopause (Aragão et al., 2011), although some authors reported a decrease in central adiposity in women after the age of 75 (Perissinotto et al., 2002). Our research revealed that cardiorespiratory fitness was positively affected by the SMI and was aggravated by high levels of adiposity, especially central, in postmenopausal women. In a previous work (Aragão et al., 2011), we showed that combining the deteriorating condition of the muscle with increased central adiposity in this population jeopardized VO_2max_, regardless of age, menopause time and the BMR.

The atherosclerotic risk generated by estrogen deprivation is related to limited production of vasodilators released by the endothelium (nitric oxide and prostacyclin) (Weiner et al., 1994) together with the limited capacity to inhibit migration and proliferation of smooth muscle cells of blood vessels. This situation is aggravated with increased visceral adipose tissue, derived from the development of an improper lipid and lipoprotein profile (Teede et al., 2010). The study of Kardassis et al. (2012) involving 4047 obese subjects also demonstrated an influence of the different components of body mass on cardiac function, revealing that lean mass was a significant predictor of change in volume of blood pumped by the ventricles in each beat and cardiac output, while an increase in fat mass was associated with disturbances in contractility and relaxation of the ventricles. The same authors reported changes in left ventricular function with increased central adiposity, reflected in an increased heart rate and blood pressure.

In the present study, the VFA and BMR proved to be independent predictors of VO_2max_, explaining 30% of its variance. The results related to the effect of HT on cardiorespiratory fitness are very contradictory in the literature, revealing differences in sample selection, products used, means of therapy administration and their starting time. Women with the VFA ≥ 100 cm^2^ had an average of −6.16 ml/kg/min compared to those whose central adiposity was considered normal, and they improved with the presence of a BMR ≥ 1238 kcal/day. Reduction of the BMR during menopause is associated with changes in body composition that occur at this stage, especially of lean tissue. Our study documented a high correlation (r ≥ 0.88) of this variable with SM and SLM of the trunk and lower limbs. An annual decline in muscle mass is particularly marked after the 5th decade, ranging between 0.6% and 2% (Roubenoff and Hughes, 2000), being more prominent in the first three years after the onset of menopause and aggravated by physical inactivity and insufficient consumption of protein and vitamin D (Maltais et al., 2009). Based on the three groups of VO_2max_ established in this study, our results show that the levels of VO_2max_ > 30.94 ml/kg/min in postmenopausal women are associated with better muscular condition and central adiposity. The combination of moderate to vigorous cardiovascular exercise with resistance exercise is very important to achieve those levels of cardiorespiratory fitness (Perez and Garber, 2011), since the first decreases the levels of fat, promotes protein synthesis and activates the production of oxidative enzymes in mitochondria, while resistance exercise increases protein synthesis in myofibrils and contributes to the reduction of visceral fat deposits. The three groups did not differ in the BMR and the characteristics of menopause.

The results suggest that women with VO_2max_ × < 26.87 ml/kg/min, when compared to the moderate and high levels, are older and have more prominent visceral adiposity. According to Church et al. (2009), regardless of the amount of weight loss, postmenopausal women may reduce central adiposity, performing at least 50% of the exercise recommendations proposed by the U.S. Department of Health and Human Services, referred to as at least 150 minutes of moderate physical activity or 75 minutes of vigorous activity per week or an equivalent combination of these two types of intensity (HHS, 2008). The physical activities should also include balance exercises in order to reduce falls (HHS, 2008).

There are some limitations of this study. The sample, which was comprised of only healthy volunteers, may not represent the entire population of PW, considering that the latter usually includes a substantial proportion of less healthy individuals. Also, the number of participants aged 65 and above was small and thus, our results cannot be extrapolated for older women. Despite the validity of bioimpedance octopolar InBody (Malavolti et al., 2003; Medici et al., 2005), which has already been reported, studies continue to show inconsistent results (Gibson et al., 2008; Volgyi et al., 2007), justifying the inclusion of other and more accurate methods for assessing total and localized body composition.

In conclusion, our data suggest that regardless of age and characteristics of menopause, the VFA and BMR significantly influence the variation of cardiorespiratory fitness of PW. Presence of VO_2max_ levels > 30.94 ml/kg/min is associated with less fat and improved muscle condition in this population. Postmenopausal women with VO_2max_ < 26.87 ml/kg/min tend to be older and show a greater VFA.

## Figures and Tables

**Table 1:** Characteristics of the sample (n=208).

**Variables**	**Mean±DPP**	**Range**
Age (years)	57.57±6.62	40.60 – 79.58
Body height (cm)	155.09 ± 5.39	142.00 – 170.00
Body mass (kg)	68.90±11.59	45.80 – 108.70
Fat mass (kg)	27.74±8.71	9.10 – 55.70
Fat mass (%)	39.34±6.92	17.90 – 53.00
Visceral fat area (cm^2^)	133.61±27.28	52.10 – 206.10
Fat-free mass (kg)	41.17±4.85	30.40 – 54.40
Skeletal muscle mass (kg)	22.40±2.93	15.80 – 30.40
Skeletal muscle mass index (%)	32.89±3.78	25.66 – 45.98
Soft lean mass arms (kg)	4.52±0.79	2.93 – 7.04
Soft lean mass index of arms (%)	6.59±0.69	4.75 – 9.20
Soft lean mass trunk (kg)	19.53±2.43	14.30 – 27.40
Soft lean mass index of trunk (%)	28.64±2.66	22.08 – 38.49
Soft lean mass legs (kg)	11.86±1.84	4.54 – 17.95
Soft lean mass index of legs (%)	17.41±2.34	4.88 – 25.03
Basal metabolic rate (kcal/day)	258.76±104.72	127.00 – 1545.00
Maximal oxygen uptake (ml/kg/min)	29.14±5.68	16.39 – 48.31

**Table 2:** Influence of body composition (%FM, FFM, VFA, SMI, SLMI_A_, SLMI_T_ and SLMI_L_) in the variation of cardiorespiratory fitness (VO_2max_), with controlling for age, basal metabolic rate and characteristics of menopause (time, nature and hormone therapy).

Dependent Variable	Independent Variables	β	R^2^ × 100	SEE
Maximal oxygen uptake (ml/kg/min)	Visceral fat area (cm^2^) Basal metabolic rate (kcal/day)	−0.563^*^ 0.165^*^	30%	4.76

**FM**, fat mass; **FFM**, fat-free mass; **VFA**, visceral fat area; **SMI**, skeletal muscle mass index; **SLMI**_A_, soft lean mass index of arms **SLMI**_T_, soft lean mass index of trunk; **SLMI**_L_, soft lean mass index of legs;

* p≤0.01

**Table 3:** Comparisons between the mean values of the visceral fat area (VFA< 100 cm^2^ and VFA≥ 100 cm^2^) and basal metabolic rate (BMR< 1238 kcal/day and BMR≥ 1238 kcal/day).

Variables	**Cut points**	**Number of Subjects**	**Mean±DP**	**Difference**
Visceral Fat Area	< 100 cm^2^	21	34.69±5.30	6.16±1.24^*^
	≥ 100 cm^2^	187	28.52±5.39	
Basal Metabolic Rate	< 1238 kcal/day	103	29.05±5.60	−0.18±0.79^*^
	≥ 1238 kcal/day	105	29.23±5.78	

* p≤0.01

**Table 4:** Comparison of age, body composition, characteristics of menopause and basal metabolic rate for the three groups of cardiorespiratory fitness.

**Variables**	**Groups of Cardiorespiratory Fitness (ml/kg/min)**			**Anova or Kruskal-Wallis**	**Tendency**		**Bonferroni**

	**LOW (< 26.87; n=69)**	**MODERATE (26.87 – 30.94; n=70)**	**HIGH (> 30.94; n=69)**				
**Age (years)**	60.16 ± 6.86	57.11 ± 6–53	55.44 ± 5.62	F= 19.02 (p<0.01)	↓	**a**	3.05±1.08 (p=0.02)
						**b**	4.72±1.08 (p<0.01)

**Fat Mass (%)**	42.39 ±6.52	39.95 ±6.01	35.67±6.52	F= 38.33 (p<0.01)	↓	**b**	6.72±1.09 (p=0.00)
						**c**	4.28±1.08 (p<0.01)

**Skeletal Muscle Mass Index (%)**	31.26±3.49	32.43 ±3.25	35.00± 3.64	F= 40.35 (p<0.01)	↑	**b**	−3.74± 0.59 (p<0.01)
						**c**	−2.57± 0,59 (p<0.01)

**Visceral Fat Area (cm^2^)**	148.39 ±24.78	133.46 ±23.29	118.97 ±25.72	F= 49.31 (p<0.01)	↓	**a**	14.93±4,17 (p<0.01)
						**b**	29.42±4,19(p<0.01)14.
						**c**	49± 4.17 (p<0.01)

**Soft Lean Mass Index Arms (%)**	6.37 ± 0.61	6.46 ± 0.62	6.92 ± 0.71	F= 24.61 (p<0.01)	↑	**b**	−0.55± 0.11 (p<0.01)
						**c**	−0.46± 0.11 (p<0.01)

**Soft Lean Mass Index Trunk (%)**	27.53±2.53	28.31±2.31	30.09 ±2.51	F= 37.54 (p<0.01)	↑	**b**	−2.56± 0.42 (p<0.01)
						**c**	−1.78± 0.42 (p<0.01)

Soft Lean Mass Index Legs (%)	16.46 ±2.36	17.28±2.12	18.48±2.09	F= 29.35 (p<0.01)	↑	**b**	−2.02± 0.37 (p<0.01)
						**c**	−1.20± 0.37 (p<0.01)

**Basal Metabolic Rate (kcal/day)**	1267,88 ±12, 81	1244.99±11. 82	1263.62 ±13.10	X^2^=0.84 (p=0.66)	-		---
					-		
					-		

**Time of Menopause (0–3)**	2.16±0.10	1.94±0.09	1.86±0.09	X^2^=5.59 (p=0.06)	-		---
					-		
					-		

**Nature of Menopause (0–1)**	1.21±0.05	1.24±0.05	1.28±0.05	X^2^=0.63 (p=0.73)	-		---
					-		
					-		

**Hormonal Therapy (0–1)**	0.52±0.06	0.53 ±0.06	0.59±0.06	X^2^=0.89 (p=0.64)	-		---
					-		
					-		

Differences between groups: **a** LOW - MODERATE; **b** LOW - HIGH; **c** MODERATE – HIGH
